# Influenza A(H10N7) Virus in Dead Harbor Seals, Denmark

**DOI:** 10.3201/eid2104.141484

**Published:** 2015-04

**Authors:** Jesper S. Krog, Mette S. Hansen, Elisabeth Holm, Charlotte K. Hjulsager, Mariann Chriél, Karl Pedersen, Lars O. Andresen, Morten Abildstrøm, Trine H. Jensen, Lars E. Larsen

**Affiliations:** Technical University of Denmark, Frederiksberg, Denmark (J.S. Krog, M.S. Hansen, E. Holm, C.K. Hjulsager, M. Chriél, K. Pedersen, L.O. Andresen, L.E. Larsen);; Anholt Gartneri & Naturpleje, Anholt, Denmark (M. Abildstrøm);; Aalborg Zoo/Aalborg University, Aalborg, Denmark (T.H. Jensen)

**Keywords:** Influenza A virus, H10N7, Phoca vitulina, viruses, harbor seals, Denmark, influenza

## Abstract

Since April 2014, an outbreak of influenza in harbor seals has been ongoing in northern Europe. In Denmark during June–August, 152 harbor seals on the island of Anholt were found dead from severe pneumonia. We detected influenza A(H10N7) virus in 2 of 4 seals examined.

Influenza A virus is widespread and affects a wide range of species, including humans (*1*). Waterfowl are considered the natural reservoir for most subtypes of influenza A virus, and most mammalian-adapted viruses initially originated in interspecies transmission from aquatic birds (*2*). Avian influenza A virus (AIV) replicates primarily in the intestinal tract of birds and is transmitted mainly through the fecal–oral route (*1*). Pinnipeds (e.g., seals) share the same shoreline habitats as many waterfowl species and therefore can be exposed to AIV. Several instances of interspecies transmission between birds and harbor seals (*Phoca vitulina*) with AIV subtypes H7N7, H4N5, H4N6, H3N8, and H3N3 have been reported in the United States, and antibodies against a wide range of subtypes have been identified in Europe, Asia, and South America (reviewed by White [*3*]). Human infections with seal influenza A virus have occasionally been reported (*3*). More recently, A(H1N1)pdm09 virus was isolated from elephant seals (*Mirounga angustirostris*) off the central coast of California, USA (*4*). To our knowledge, AIV in harbor seals off the coast of northern Europe was first reported in April 2014 (*5*).

## The Study

**Figure 1:**
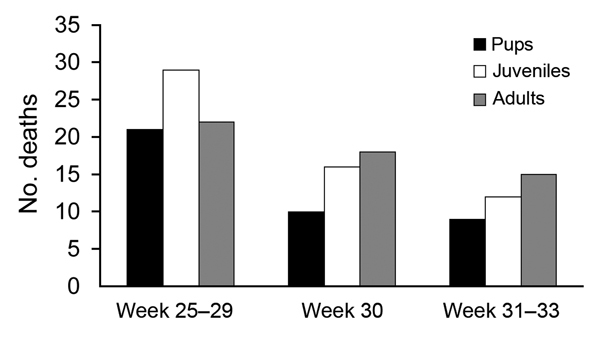
Deaths of harbor seals (*Phoca vitulina*) on the small island of Anholt, Denmark, summer 2014.

During June 16–August 13, 2014, a total of 152 harbor seals were found dead on the shore of the small island of Anholt in Denmark. A few carcasses were reported in late June, and deaths peaked in mid-July (Figure 1). Four freshly or recently dead harbor seals were submitted to the National Veterinary Institute, Technical University of Denmark, for necropsy and laboratory examination. The seals were juveniles; 3 were males; and the animals’ body conditions were normal or slightly below normal (2 seals each). All had intense reddening of the lungs, with multifocal condensation of lung tissue, moderate to massive amounts of blood in thorax, and an intensely hyperemic trachea. One seal also had blood in the mouth. No lesions were apparent in other organs, but all 4 animals had empty stomachs. In all seals, histopathologic examination of the lungs showed massive suppurative and necrotizing bronchopneumonia and many bacteria in the alveoli. In the interstitial tissues, edema, fibrin, and neutrophils were seen. Samples from relevant organs were tested for bacteria on selective and nonselective culture media. Samples from the lungs revealed massive growth of *Pseudomonas aeruginosa* in all animals, indicating severe bacterial pneumonia (*6*). In addition, variable growth of *Streptococcus equi* subsp. *zooepidemicus* was found.

We tested samples of lung and spleen for influenza A virus by a slightly modified version of a previously described real-time reverse transcription PCR (RT-PCR) that targeted the matrix gene (*7*) and for phocine and canine distemper virus by 1-step RT-PCR using previously described primers that targeted the P (phosphoprotein) gene (*8*). All 4 samples were negative for distemper virus. Samples from lungs and spleen from 2 seals were positive for influenza A virus (Table). RNA from the influenza A virus–positive samples subsequently tested negative for H5 and H7 subtypes by subtype-specific real-time RT-PCR.

**Table:** Results of PCR and sequence analysis of the 2 influenza A virus–positive harbor seals, Denmark, 2014*

Virus	Tissue	Real-time RT-PCR, M (C_t_)	Sequence	
			Hemagglutinin	Neuraminidase
A/harbor seal/Denmark/14–5060–1lu/2014	Lung	Positive (32.9)	Negative	Positive
A/harbor seal/Denmark/14–5060–1sp/2014	Spleen	Positive (37.3)	Negative	Negative
A/harbor seal/Denmark/14–5061–1lu/2014	Lung	Positive (27.8)	Positive	Positive
A/harbor seal/Denmark/14–5061–1sp/2014	Spleen	Positive (38.2)	Negative	Negative
*C_t_, cycle threshold; M, matrix; RT-PCR, reverse transcription PCR.				

**Figure 2:**
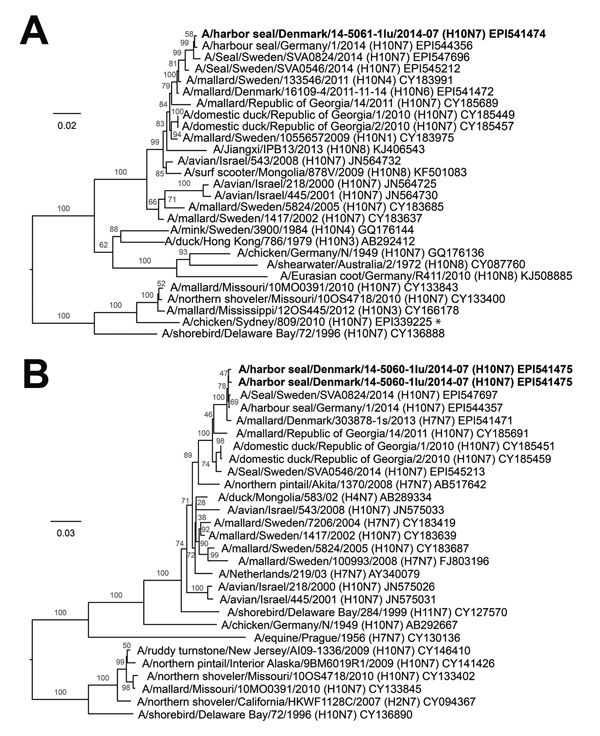
Phylogenetic trees of selected avian influenza A virus sequences. Boldface indicates sequences identified from harbor seals in Denmark during 2014. A) H10 avian influenza A virus sequences. The asterisk denotes the H10N7 subtype that also caused disease in humans (*9*). B) N7 avian influenza A virus sequences. Sequences were aligned with CLC Main Workbench version 7.02 (CLC bio, Aarhus, Denmark) by using the MUSCLE algorithm, and phylogenetic trees were constructed by using the neighbor-joining method with 1,000 bootstrap replicates. Only bootstrap values >75% are shown. Scale bars indicate mutations per site.

Full length hemagglutinin (HA) and neuraminidase (NA) genes were amplified using RT-PCR on the RNA extracted from the spleen and lung. Sequences of HA and NA were only obtained from lung samples and used in the following phylogenetic analysis. We could not sequence the spleen samples because of low viral loads (Table). Using BLAST search (http://blast.ncbi.nlm.nih.gov/Blast.cgi) and phylogenetic analysis, we identified the subtype as H10N7. The highest nucleotide similarity of HA gene from the seal to AIVs was to A/mallard/Sweden/133546/2011(H10N4) (98.4%) and a wild bird AIV from Denmark A/mallard/Denmark/16109-4/2011(H10N6) (98.3%). The NA segments from the harbor seals were 99.5% identical to each other and 99.3% identical to an AIV isolated from a commercial flock of game birds in Denmark in 2013 (A/mallard/Denmark/303878-1s/2013(H7N7)) and 97.6% identical to A/domestic duck/Republic of Georgia/1/2010(H10N7). In general, both the HA (Figure 2, panel A) and NA (Figure 2, panel B) segments showed high-level nucleotide sequence identity to AIVs from birds sampled in Scandinavia and the Republic of Georgia. Detection of the H10N7 virus in seals has been reported from Denmark, Germany, the Netherlands, and Sweden. The seal influenza virus was initially collected in Sweden in April (*5*); this isolate is 98.7% nt (HA) and 97.0% nt (NA) identical to the strain from Denmark. The samples collected from Germany and Sweden in late August are more similar to the samples from Denmark, with identities of 99.2%–99.7% nt; the strains from Denmark and Germany are most similar. At the amino acid level, the HA of the viruses from Denmark and Germany was 99.3% identical, whereas the early strain from Sweden was only ≈97.5% identical to all the other strains. The most recent reported strain from Sweden was 98.5% aa identical to the Denmark and Germany strains. The lower amino acid identity of HA reflects the large proportion of nucleotide mutations, which results in amino acid changes (dN/dS = 0.7) indicating that adaptation to seals is in progress. Further analysis of the HA sequence showed that the amino acids in the receptor binding site were conserved compared to other H10 sequences (*10*). The predicted amino acid sequence of the HA cleavage site of all the A (H10N7) seal viruses were unique (PELVQGR*GLF) and contained only 1 basic amino acid, which indicated low pathogenicity.

## Conclusions

Outbreaks of influenza A virus in marine mammals have been repeatedly described in North America and Asia and have often been linked to increased deaths and severe lung lesions (*11*–*14*), as in the outbreak reported here (*5*). The pathologic findings are consistent with findings described in previous outbreaks in seals, but the histopathologic findings indicate severe secondary bacterial pneumonia, which was also confirmed by cultures of several different bacteria. Thus, the severity of infection probably was caused by AIV infection combined with secondary bacterial infections, which also explain why experimentally reproducing severe disease in seals has been difficult (*14*).

The influenza A (H10N7) virus that we detected probably was of avian origin, as indicated by its high similarity to contemporary AIV isolates. The predicted amino acid sequence of the HA cleavage site indicated low pathogenic AIV because it did not contain multiple basic amino acids. However, because the amino acid sequence of the cleavage site is unique and because the virus also was detected in the spleens, systemic spread and a highly pathogenic phenotype of the virus cannot be ruled out. Indeed, influenza A virus of the H10 subtype has been shown to have characteristics of highly pathogenic AIV in chickens, despite the lack of multiple basic amino acids at the cleavage site (*15*). Furthermore, some strains of H10 have proved to be highly pathogenic in mink (*10*).

During the current outbreak, we found no epidemiologic evidence of human exposure, but human infection has been reported in connection with previous outbreaks of influenza A virus in harbor seals (*3*). Furthermore, another A(H10N7) virus, A/chicken/Sydney/809/2010(H10N7), from chicken infected workers in a poultry abattoir in Australia was of US lineage and thus differed significantly (81.0% identity) from the harbor seal HA in our current study (Figure 2, panel A) (*9*). Thus, whether humans are at risk for infection with the Denmark A(H10N7) from contact with seals infected with influenza A(H10N7) virus is unclear.
